# The Effect of 600 mg Alpha-lipoic Acid Supplementation on Oxidative Stress, Inflammation, and RAGE in Older Adults with Type 2 Diabetes Mellitus

**DOI:** 10.1155/2019/3276958

**Published:** 2019-06-12

**Authors:** Víctor Manuel Mendoza-Núñez, Beatriz Isabel García-Martínez, Juana Rosado-Pérez, Edelmiro Santiago-Osorio, José Pedraza-Chaverri, Vicente Jesús Hernández-Abad

**Affiliations:** ^1^Research Unit on Gerontology, FES Zaragoza, National Autonomous University of Mexico, Mexico City, Mexico; ^2^Hematopoiesis and Leukemia Laboratory, Research Unit on Cell Differentiation and Cancer, FES Zaragoza, National Autonomous University of Mexico, Mexico City, Mexico; ^3^Department of Biology, Faculty of Chemistry, National Autonomous University of Mexico (UNAM), Mexico City 04510, Mexico; ^4^Pharmaceutical Research Laboratory, FES Zaragoza, National Autonomous University of Mexico, Mexico City, Mexico

## Abstract

Alpha-lipoic acid (ALA) has been used as a dietary supplement at different doses in patients with diabetes mellitus type 2 (T2DM) due to its antioxidant, anti-inflammatory, and hypoglycemic effects. However, the reports on the effects of ALA are controversial. For this reason, the purpose of the present study was to determine the effect of 600 mg/day of ALA on the markers of oxidative stress (OxS) and inflammation and RAGE in older adults with T2DM. A quasiexperimental study was carried out with a sample of 135 sedentary subjects (98 women and 37 men) with a mean age of 64 ± 1 years, who all had T2DM. The sample was divided into three groups: (i) experimental group (EG) with 50 subjects, (ii) placebo group (PG) with 50 subjects, and control group (CG) with 35 subjects. We obtained the following measurements in all subjects (pre- and posttreatment): glycosylated hemoglobin (HbA1c), receptor for advanced glycation end products (RAGE), 8-isoprostane, superoxide dismutase (SOD), glutathione peroxidase (GPx), total antioxidant status (TAS), and inflammatory (CRP, TNF-*α*, IL-6, IL-8, and IL-10) markers. Regarding the effect of ALA on HbA1c, a decrease was observed in the EG (baseline 8.9 ± 0.2 vs. posttreatment 8.6 ± 0.3) and the PG (baseline 8.8 ± 0.2 vs. posttreatment 8.4 ± 0.3) compared to the CG (baseline 8.8 ± 0.3 vs. six months 9.1 ± 0.3) although the difference was not statistically significant (*p* < 0.05). There was a statistically significant decrease (*p* < 0.05) in the blood concentration of 8-isoprostane in the EG and PG with respect to the CG (EG: baseline 100 ± 3 vs. posttreatment 57 ± 3, PG: baseline 106 ± 7 vs. posttreatment 77 ± 5, and CG: baseline 94 ± 10 vs. six months 107 ± 11 pg/mL). Likewise, a statistically significant decrease (*p* < 0.05) in the concentration of the RAGE was found in the EG (baseline 1636 ± 88 vs. posttreatment 1144 ± 68) and the PG (baseline 1506 ± 97 vs. posttreatment 1016 ± 82) compared to CG (baseline 1407 ± 112 vs. six months 1506 ± 128). A statistically significant decrease was also observed in all markers of inflammation and in the activity of SOD and GPx in the CG with respect to the EG and PG. Our findings suggest that the administration of ALA at a dose of 600 mg/day for six months has a similar effect to that of placebo on oxidative stress, inflammation, and RAGE in older adults with T2DM. Therefore, higher doses of ALA should be tried to have this effect. This trial is registered with trial registration number ISRCTN13159380.

## 1. Introduction

Oxidative stress (OxS) is a biochemical imbalance that is propitiated by excessive production of reactive oxygen and nitrogen species, which provoke oxidative damage to biomolecules and cannot be counteracted by antioxidative systems. This is an important factor that contributes to aging and the development of several diseases, including type 2 diabetes mellitus (T2DM) [1, 2]. For several decades, it has been shown that OxS and the chronic inflammatory process are involved in the physiopathological mechanisms of T2DM [3]. In this sense, the chronic hyperglycemia that is present in T2DM activates several unusual metabolic pathways in organisms, such as the sorbitol pathway (or that of aldose reductase), nonenzymatic protein glycosylation, glucose autooxidation, modification of protein kinase C activity, pseudohypoxia, lipoprotein-altered metabolism, and cytokine-associated alteration. All these pathways generate reactive oxygen species (ROS) and, consequently, OxS [4]. Likewise, several studies have shown that aging and/or T2DM increases the synthesis and secretion of cytokines, such as interleukin 6 (IL-6), tumor necrosis factor-alpha (TNF-*α*), and free radicals. These are all recognized as factors that increase the risk of disease-related complications [4, 5]. In this regard, our research group showed that aging in the context of diabetes increases the production of OxS and causes inflammation [6].

For this reason, some therapeutic supplements have been proposed with antioxidant and anti-inflammatory properties, such as vitamins A, C, E; omega 3 and 6 fatty acids; coenzyme Q10; melatonin; and alpha-lipoic acid [7–9].

Alpha-lipoic acid (ALA) is an amphipathic substance, which is synthesized in the mitochondria of plants and animals from octanoic acid and cysteine as a sulfur donor through the reactions catalyzed by the enzyme lipoic acid synthase. The participation of ALA in oxidative metabolism is essential. [7] ALA chemically exists in two enantiomeric forms R and S although only the R isoform acts as a cofactor in the oxidant metabolism, since it binds through an amide bond to the amino group of the lysine residues. This allows it to form a lipoamide, which is a cofactor of the enzymes, pyruvate dehydrogenase and *α*-ketoglutarate dehydrogenase [10, 11].

Several studies have demonstrated the antioxidant, anti-inflammatory, and hypoglycemic properties of ALA [12–15]. Furthermore, ALA has been shown to have a positive effect on the OxS linked with aging [16]. For this reason, the aim of the present study was to determine the effect of 600 mg/day of ALA on some markers of OxS and inflammation and RAGE in older adults with T2DM.

## 2. Materials and Methods

### 2.1. Design and Subjects

A quasiexperimental study was carried out with a sample of 135 sedentary subjects (98 women and 37 men) with a mean age of 64 ± 1 years, who all had T2DM. The age range of the subjects was 60–74 years. All participants gave their written informed consent for the inclusion in the study. The investigation protocol was approved by the Ethics Committee of the Universidad Nacional Autónoma de México (UNAM) Zaragoza Campus (25/11/SO/3.4.3). It was also registered in International Standard Randomised Controlled Trial Number (ISRCTN13159380) [17]. We have used some protocols and standardized techniques by our research group [18].

### 2.2. Inclusion Criteria

All subjects were independent and had a medical diagnosis of type 2 diabetes mellitus for one to three years without complications or comorbidity and were under medical treatment in a public hospital: (i) body mass index < 35; (ii) medicines: all patients taking two tablets of glibenclamide/metformin (5/500 mg) per day as a hypoglycemic treatment and not taking antioxidant supplements (vitamins or minerals) nor anti-inflammatory drugs for at least 6 months prior to initiation of or during the study; (iii) habits: no smoking, no frequent alcoholic drinks (less than two drinks or beers per week), or no drug addictions (marijuana, cocaine and others) in the last three years; and (iv) sedentary: all participants who reported that they do not exercise regularly during the last year.

### 2.3. Study Sample

We invited 200 older adults who were under medical supervision for type 2 diabetes mellitus in a public hospital in Mexico City. In this regard, 47 did not meet the inclusion criteria and 18 did not agree to participate in the study, for the reason that they did not have time for the follow-up meetings every four weeks for the delivery of the medication, self-report of health status, registration of secondary reactions, and reinforcement for therapeutic adherence.

**Figure 1:**
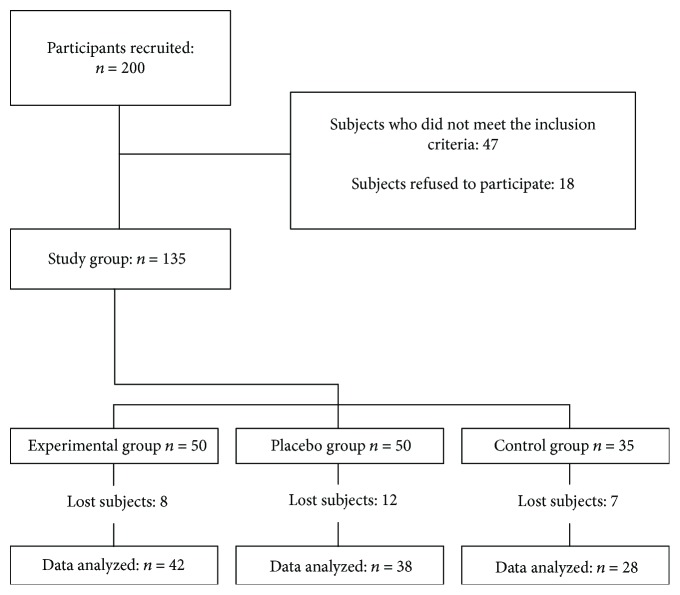
Outline of the study.

In Figure 1, we outlined the study. All participants were assigned to the following study groups: (i) the experimental group (EG) with 50 individuals, (ii) the placebo group (PG) also with 50 individuals, and the control group (CG) with only 35 individuals. The assignment of the EG and PG was randomized. EG received 600 mg of racemic alpha-lipoic acid (two capsules of 300 mg per day), PG received two capsules containing microcrystalline cellulose (295 mg) plus magnesium stearate (5 mg) of pharmaceutical presentation similar to that of the treatment, and CG did not receive any treatment. Alpha-lipoic acid and placebo capsules were manufactured by ProductosMedix®. Also, the control group (CG) with 35 subjects was added for the aim of assessing the placebo effect.

The medication contained in the treatment capsules provided by ProductosMedix® was analyzed by polarimetry, obtaining a specific rotation of 0° (the mixture contained 50 : 50 of R and S enantiomers).

The treatment was self-administered orally in a daily base. The EG and PG were instructed to record adverse reactions. We had the same personal contact with the three groups. All groups had a monthly meeting with the research team. In this meeting, the subjects were informed about a lifestyle conducive to successful aging (ageism, active aging, healthy aging, mental and social functioning, healthy feeding, physical exercise and aging, mild cognitive impairment, and depression). Likewise, the placebo and treatment groups were informed about the antioxidant effects of alpha-lipoic acid (ALA).

The following measurements were performed in the three study groups: blood pressure, anthropometric measurements, glycosylated hemoglobin (HaA1c), biochemical parameters (lipid profile, glucose, albumin, and uric acid), blood concentration of 8-isoprostane, superoxide dismutase and glutathione peroxidase of red blood cells, inflammatory cytokines, receptor of advanced glycation end products (RAGE), and plasma concentration of ALA. All measurements were performed at baseline and after six months.

### 2.4. Dietary Intake

All participants were given a guide on the amount and type of food they could consume: recommended consumption of 2500 kcal per day. In this sense, all kilocalories of 50% carbohydrate consumption, 30% fat, and 20% protein were considered, in addition to an intake of 1500 mL of water. Regarding carbohydrates, the foods to be eaten per day were 3 tortillas/boiled rice or pasta and, regarding proteins, fish and chicken (daily) and red meats twice a week. Daily consumption of boiled and raw vegetables (chayote, broccoli, squash, green beans, and carrots) was recommended. The fruits allowed were papaya, melon, apple, oranges, guavas, strawberries, and grapes. The prohibited foods were all kinds of bread, soft drinks, sugar (natural and artificial sweeteners), and fruit juices or canned juices. Each month, they were insisted on the importance of following the guide of food consumption that was provided to them.

The caloric intake was measured by 24 h dietary total recall [19].

### 2.5. Anthropometric Measurements

The anthropometric measurements of weight, height, and waist circumference were obtained following a standardized protocol after recording the clinical history and conducting the physical examination. Weight was measured while the subject was wearing underwear and a hospital smock and was in a fasting state (after evacuation). A Torino® scale (Tecno Lógica Mexicana, Mexico City, Mexico), calibrated before each measurement, was used. Height was obtained with an aluminum cursor stadiometer graduated in millimeters. The subject was barefoot with the back and head in contact with the stadiometer in the Frankfurt horizontal plane. The body mass index (BMI) was calculated by dividing weight (kg) by height squared (m^2^). Waist circumference (cm) was measured to the nearest 0.5 cm with a tape measure at the umbilical scar level [18].

### 2.6. Blood Pressure

Blood pressure (BP) was measured following the standardized protocol according to the Official Mexican Norm (Norma Official Mexicana) [20]. Using a mercurial manometer at both arms, BP was registered before the treatment (BP Baseline) and six months after (BP six months); measurements were taken in the morning in a fasted condition or 2 hours after breakfast in the sitting and standing positions. Subjects with pseudohypertension were identified by applying the Osler technique (feeling the radial pulse when the manometer registered values above the true systolic pressure). Blood pressure was taken by medical technicians who attended training sessions to standardize the procedures. The technicians were supervised to avoid possible biases in measurement.

### 2.7. Blood Sampling and Biochemical Analyses

Blood samples were collected before and after the treatment at six months in the three groups (EG, PG, and CG) by venipuncture after a 10 h fasting period and placed in vacutainer/siliconized test tubes without anticoagulant for biochemical determinations (glucose, urate, albumin, lipid profile, and inflammatory cytokines) and with heparin for glycosylated hemoglobin determination and oxidative stress tests.

Glucose, urate, albumin, cholesterol, triglycerides, and HDL-C concentration levels were determined using a Merck Vitalab Eclipse autoanalyzer (Merck, Dieren, The Netherlands). In particular, glucose levels were measured by the glucose oxidase method, and urate levels by the uricase colorimetric method. Albumin levels were measured with the bromocresol green technique.

The low-density lipoproteins (LDL cholesterol) were calculated by the Friedewald equation: LDL = total cholesterol − (triglycerides/5 + HDL)[21]. The C-reactive protein was measured in serum by immunoturbidimetric assay.

Glycosylated hemoglobin (HbA1c) was measured in a whole blood sample with an immunoturbidimetric assay with an automated Selectra Junior clinical chemistry analyzer.

High and normal control sera were included as quality controls (Randox Laboratories Ltd.). The intra- and interassay variation coefficients were less than 5% for all determinations.

### 2.8. Isoprostane Blood Concentration

8-Isoprostane level was measured in plasma samples using ELISA (Cayman, USA) following the manufacturer's instructions (Catalog No. 516351). This assay is based on the competition between 8-isoprostane and an 8-isoprostane-acetylcholinesterase (AChE) conjugate (8-Isoprostane Tracer) for a limited number of 8-isoprostane-specific rabbit antiserum binding sites. Because the concentration of the 8-Isoprostane Tracer is held constant while the concentration of 8-isoprostane varies, the amount of the 8-Isoprostane Tracer that is able to bind to the rabbit antiserum will be inversely proportional to the concentration of 8-isoprostane in the well. This rabbit antiserum-8-isoprostane (either free or tracer) complex binds to the rabbit IgG mouse monoclonal antibody that has been previously attached to the well. The plate is washed to remove any unbound reagents, and then, Ellman's Reagent (which contains the substrate to AChE) is added to the well. The product of this enzymatic reaction has a distinct yellow color and absorbs strongly at 412 nm. The intensity of this color, determined spectrophotometrically, is proportional to the amount of 8-isoprostane.

### 2.9. Plasma Total Antioxidant Status

Plasma total antioxidant status levels were quantified using 2,2′-azino-bis(3-ethylbenzthiazoline-6-sulfonic acid) (ABTS) (Randox Laboratories Ltd.), which is incubated with a peroxidase to produce the radical cation ABTS^+^. The bluish green staining of the ABTS^+^ cation is relatively stable and measured at 600 nm; antioxidants present in the plasma cause the suppression of this color production to a degree that is proportional to the concentration. The kinetics reaction was measured with a Shimadzu UV-1601 spectrophotometer (Shimadzu, Kyoto, Japan).

### 2.10. Red Blood Cell Superoxide Dismutase

In this method, superoxide radicals are generated by employing xanthine and xanthine oxidase. The formed radical reacts with 2-(4-iodophenyl)-3-(4-nitrophenol)-5-phenyltetrazolium chloride to form a red formazan color, which is measured at 505 nm. The superoxide dismutase (SOD) in the sample causes the inhibition of this reaction; the SOD activity is proportional to the degree of inhibition of the reaction (Randox Laboratories Ltd.). Kinetics were measured with a Shimadzu UV-1601 spectrophotometer (Shimadzu Kyoto, Japan).

### 2.11. Red Blood Cell Glutathione Peroxidase

The glutathione peroxidase (GPx) catalyzes glutathione (GSH) oxidation by cumene hydroperoxide. In the presence of glutathione reductase (GR) and nicotinamide adenine dinucleotide phosphate (NADPH), oxidized GSH is immediately converted into the reduced form with a concomitant oxidation of NADPH to NADP^+^ (Randox Laboratories Ltd.). The decrease in absorbance is measured at 340 nm; we used a Shimadzu UV-1601 spectrophotometer (Shimadzu).

We calculated the SOD/GPx ratio and the antioxidant gap (AOGAP) using the following equation: AOGAP = (TAS‐[(albumin(mmol) × 0.69) + uric acid(mmol)]) [22]

### 2.12. Receptor for Advanced Glycation End Products (RAGE)

RAGE concentrations were measured by RAGE (DRG00, Quantikine; R&D System Inc., Minneapolis, MN, USA) according to the manufacturer's protocol. For this assay, a monoclonal antibody for human RAGE is used, which has been bound to the bottom of each well. After the correct addition of reagents, standards, and samples, the RAGE-anti RAGE reaction occurs, and after the corresponding incubations and washings, the optical density of the complex formed is read in a microplate reader set at 450 nm. The RAGE concentration is directly proportional to the intensity of the color developed by the RAGE-substrate complex.

### 2.13. Inflammatory Cytokines and C-reactive Protein (CRP)

Aliquots of serum sample were assayed by flow cytometry (CBA Kit, Human Inflammatory Cytokine, BD) to determine the levels of interleukin (IL)-1*β*, IL-6, IL-8, IL-10, and tumor necrosis factor-alpha (TNF-*α*) [23]. For the measurement of CRP, particles coated with anti-human CRP antibodies were used, which were agglutinated by CRP molecules present in the serum samples analyzed. Since the agglutination causes changes in the absorbance proportionally to the concentration of CRP and after comparison with a calibrator, it was possible to determine the exact concentration of the protein. The test was carried out on the Selectra Junior-automated equipment, under a turbidimetric principle, using a commercial kit from Spinreact (CRP TURBI 1107101L).

### 2.14. Measurement of ALA Plasma Concentration

ALA was quantified by high-performance liquid chromatography (HPLC) coupled to electrochemical detection. 1 mL of acetonitrile was added to each sample and centrifuged at 3000 rpm for 5 minutes to precipitate the proteins. The supernatant was separated and placed into a solid phase extraction cartridge (Hypersep, Thermo Scientific™). Then, the supernatant was washed with 3 mL of methanol followed by 2 mL of acetate buffer solution (0.01 M, pH = 4). The eluate was collected into a clean tube and dried under nitrogen stream at room temperature. The residue was reconstituted with 1 mL of mobile phase (methanol/0.01 M acetate buffer (pH = 4) (60 : 40 *v*/*v*) and injected into the chromatograph (KnauerSmartline) coupled to an electrochemical detector (Recipe EC3000) in a direct current (DC) mode at the detector potential of 1000 mV under the following conditions: mobile phase pumped at a flow rate of 1 mL·min^−1^, temperature of 25°C, and pressure of 1500 PSI. The chromatographic separation was carried out on a Hypersil Gold C18 column of 150 mm × 4.5 mm and 5 *μ*m particle size (Thermo Scientific ™). A chromatogram to each sample was obtained, and the area under curve was calculated. Afterwards, all areas under the curve obtained from samples were interpolated in a standard curve to calculate ALA concentration [24].

### 2.15. Statistical Analysis

The statistical analysis of the data was done as follows: First, a descriptive analysis was performed to calculate the means and standard error (SE) of the outcomes over time. Second, a repeated measure multivariate analysis of variance was conducted to investigate the effects of the ALA on the changes of biochemical parameters, oxidative stress, and proinflammatory markers over time. The between-subject factor was each group (i.e., the experimental group versus the placebo and control groups), and the within-subject factor was time (baseline versus six months) [25], after Dunnett's post hoc test was done. Likewise, a correlation analysis between ALA concentration with oxidative stress and inflammation markers and RAGE was carried out. In the present study, the significance level (*α*) was set at <0.05 for all statistical analyses. We used the statistical analysis program IBM SPSS Statistics 20.0.

## 3. Results

### 3.1. ALA Plasma Concentration

**Figure 2:**
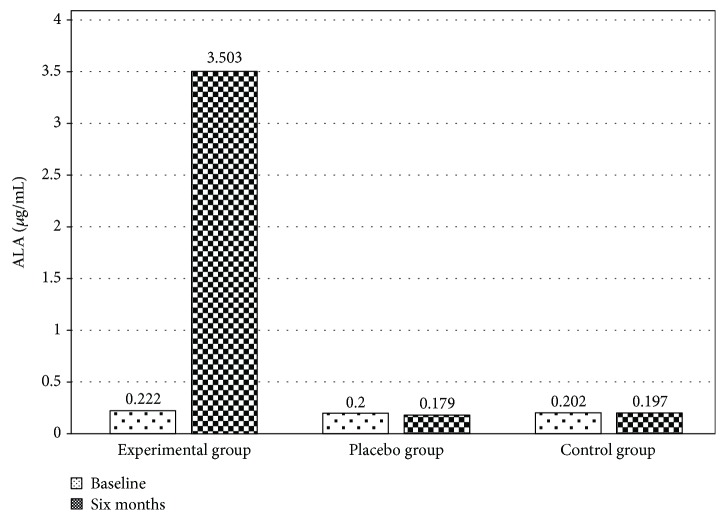
Plasma concentration of alpha-lipoic acid (ALA) before and after treatment in the study groups. A significant increase in the concentration of ALA was observed in the experimental group (before 0.222 ± 0.03 vs. after 3.503 ± 0.2*μ*g/mL) compared to the placebo (before 0.2 ± 0.02 vs. after 0.179 ± 0.03*μ*g/mL) and control groups (before 0.202 ± 0.04 vs. after 0.197 ± 0.03*μ*g/mL). The values represent mean + standard error. Repeated measure analysis of variance. *p* < 0.01.

Figure 2 shows the blood ALA concentration of the participants in the experimental group (EG) (baseline 0.222 ± 0.03 vs. posttreatment 3.503 ± 0.2*μ*g/mL), placebo group (PG) (baseline 0.2 ± 0.02 vs. posttreatment 0.179 ± 0.03*μ*g/mL), and control group (CG) (baseline 0.202 ± 0.04 vs. after six months 0.197 ± 0.03*μ*g/mL). Statistically significant differences were observed in the EG with respect to PG and CG after the treatment (*p* < 0.01).

### 3.2. Caloric Intake, Anthropometric Measurements, and Blood Pressure

All subjects had a caloric intake between 3,000 and 3,500 kcal per day pre- and posttreatment, considering 50 to 60% of carbohydrates, 30 to 40% of fats, and 20 to 30% of proteins.

The data on the body mass index and blood pressure did not show statistically significant differences between the groups after six months of treatment (*p* > 0.05) (Table 1).

### 3.3. Biochemical Parameters

Regarding the biochemical parameters, a statistically significant increase was observed in the blood HDL concentration in the EG (baseline 48 ± 1 vs. posttreatment 54 ± 2 mg/dL) and PG (baseline 46 ± 2 vs. posttreatment 51 ± 2 mg/dL) compared to the CG (baseline 64 ± 2 vs. six months 58 ± 3 mg/dL; *p* < 0.05). We also found a statistically significant increase in the concentration of total cholesterol in the CG (baseline 198 ± 10 vs. six months 215 ± 8 mg/dL) compared to the EG (baseline 180 ± 8 vs. posttreatment 180 ± 9 mg/dL) and PG (baseline 176 ± 7 vs. posttreatment 158 ± 8 mg/dL) (*p* < 0.05). Regarding the effect of ALA on HbA1c, a decrease was observed in the EG (baseline 8.9 ± 0.2 vs. posttreatment 8.6 ± 0.3) and the PG (baseline 8.8 ± 0.2 vs. posttreatment 8.4 ± 0.3) compared to the CG (baseline 8.8 ± 0.3 vs. six months 9.1 ± 0.3) although the difference was not statistically significant (*p* = 0.20). There were no significant changes in the rest of the biochemical parameters (Table 2).

### 3.4. OxS Markers and RAGE

There was a statistically significant decrease (*p* < 0.05) in the blood concentration of 8-isoprostane in the EG and PG with respect to the CG (EG: baseline 100 ± 3 vs. posttreatment 57 ± 3, PG: baseline 106 ± 7 vs. posttreatment 77 ± 5, CG: baseline 94 ± 10 vs. six months 107 ± 11 pg/mL). We also observed a statistically significant decrease in the activity of SOD in the CG (baseline 182 ± 2 vs. six months 172 ± 1 IU/L, *p* < 0.05) compared to the EG (baseline 178 ± 1 vs. posttreatment 177 ± 1 IU/L) and PG (baseline 176 ± 1 vs. posttreatment 172 ± 1 IU/L). Likewise, there was a statistically significant decrease in the activity of the GPx in the CG (baseline 9531 ± 815 vs. six months 6223 ± 613 IU/L, *p* < 0.05) compared to the EG (baseline 8409 ± 507 vs. posttreatment 9694 ± 458 UI/L) and PG (baseline 8273 ± 575 vs. posttreatment 8691 ± 355 UI/L). Consequently, these changes were reflected in a statistically significant increase (*p* < 0.05) in the SOD/GPx ratio in the CG. Likewise, a statistically significant decrease (*p* < 0.05) in the concentration of the RAGE was found in the EG (baseline 1636 ± 88 vs. posttreatment 1144 ± 68) and the PG (baseline 1506 ± 97 vs. posttreatment 1016 ± 82) compared to CG (baseline 1407 ± 112 vs. six months 1506 ± 128). There were no statistically significant differences in TAS and AOGAP (Table 3).

### 3.5. Inflammatory Cytokines and CRP

The blood concentration of all the inflammatory cytokines measured (IL-1*β*, IL-6, IL-8, IL-10, and TNF-*α*) and CRP showed a statistically significant increase in CG after six months (*p* < 0.05) with respect to the EG and PG (Table 4).

### 3.6. Correlation between ALA and OxS Markers

A positive correlation was observed between the blood concentration of ALA with the activity of the SOD (*r* = 0.279, *p* < 0.01) and the GPx (*r* = 0.249, *p* < 0.05). Furthermore, the blood concentration of ALA had a negative correlation with the concentration of 8-isoprostane (*r* = –0.247, *p* < 0.05) (Table 5).

### 3.7. Correlation between ALA and Inflammatory Markers

The blood concentration of ALA had a negative correlation with TNF-*α* (*r* = –0.250, *p* < 0.05), IL-6 (*r* = –0.249, *p* < 0.05), and IL-1*β* (*r* = –0.329, *p* < 0.01) (Table 6).

## 4. Discussion

Alpha-lipoic acid (ALA) is synthesized *de novo* in the body from fatty acid and cysteine in low quantities. Therefore, it is important to consume exogenous sources of ALA to have a therapeutic effect. In this regard, it has been shown that ALA is found abundantly in animal tissues, being located mainly in THE viscera, such as THE heart, liver, and kidneys. However, it is also found in high concentrations in vegetables, such as broccoli, spinach, tomatoes, peas, potatoes, and rice bran [26, 27]. However, to fully utilize its antioxidant, anti-inflammatory, and hypoglycemic properties, a racemic mixture available in capsules was prepared from a commercial product provided by ProductosMedix®. In this sense, it has been demonstrated that the gastrointestinal absorption of ALA depends on the timing of ingestion as there is a greater absorption of the compound if it is administered 30 minutes before or 2 hours after food intake. It has also been shown that the absorption of ALA is enantioselective since the R isoform is absorbed more efficiently than S [28–30].

Several studies have shown a therapeutic effect of ALA with doses of 300–1800 mg per day administered for three months up to four years. In this regard, we decided to administer 600 mg per day of ALA for six months in the present study, which took the evidence of effectiveness and safety for the elderly population into consideration [31–39].

Among the beneficial health effects of taking ALA supplements, it has been observed that the administration of ALA is capable of promoting the synthesis of nitric oxide. In this regard, it has been proposed that ALA stimulates the PI3K/Akt signaling pathway, with Akt being responsible for the phosphorylation and subsequent activation of eNOS (NO synthase), an enzyme that catalyzes the conversion of L-arginine and O_2_ in citrulline with NO release [9]. This is a molecule that is responsible for regulating the elasticity of the walls of blood vessels and improving endothelial function, which results in a decrease in blood pressure. In addition, ALA reduces the levels of reactive oxygen species, which also favors endothelial function and subsequently lowers blood pressure [31]. However, no statistically significant differences were observed after treatment with ALA in our study. In this sense, our findings are similar to that reported in the systematic review published by Mohammadi et al., who found that the administration of ALA at doses of 300–1800 mg/day has no effect on arterial hypertension [40].

On the other hand, it has been observed that the administration of ALA decreases the body weight and, consequently, the BMI in both animals and humans. In this regard, although the precise mechanisms are unknown, it has been suggested that ALA participates in the modulation of some pathways that are involved in energy homeostasis, the synthesis and oxidation of lipids, and the elimination of cholesterol through the liver. One of these pathways involves the protein kinase being activated by adenosine monophosphate (AMPK). It is known that AMPK integrates both hormonal and nutrient signals in the hypothalamus, which gives it a functional role in behavior that is related to food consumption and energy expenditure. Likewise, it has been suggested that ALA has an anorectic effect, which is more evident during the first two weeks of supplementation and gradually dissipates over time [41, 42]. In this regard, no statistically significant differences were observed in the BMI after treatment with ALA in our study, which is in contrast to previously reported results as researchers found that the administration of ALA induces a moderate loss of body weight and a statistically significant decrease in the BMI. However, this effect was observed with higher doses of ALA (1200 mg/day and 1800 mg/day) [42–44] compared to the 600 mg/day administered in our investigation. Likewise, it is important to point out that time is another determining factor since the anorectic effect only occurs during the first weeks as mentioned above.

Regarding the effect of ALA on lipid metabolism, it has been pointed out that it reduces lipogenesis at the peripheral level by increasing the *β*-oxidation of fatty acids and improving the energy expenditure of the whole body [45, 46]. In this sense, a statistically significant increase in blood HDL concentration was observed in the EG after treatment (*p* < 0.05) in our study although this increase was also observed in the PG, which suggests that the administration of ALA at a dose of 600 mg/day has an effect on HDL that is similar to placebo. In this regard, similar findings were reported by Khabbazi et al. in a study conducted in patients with renal failure, who were given 600 mg/day of ALA for eight weeks [47]. In addition, Koh et al. did not observe statistically significant changes in the HDL blood concentration in obese adults, who consumed 1200 and 1800 mg/day of ALA for 20 weeks in comparison with the placebo group [48]. These results are in contrast to the statistically significant increase in HDL concentration found by Zhang et al. in obese patients given a dose of 600 mg/day of ALA for two weeks compared with a placebo group [46]. For this reason, the effect of ALA on the lipid profile remains controversial so its indication for these purposes would not be justified.

On the other hand, ALA has been shown to have a hypoglycemic effect since it improves the uptake and utilization of glucose by fat cells and skeletal muscle by inducing the translocation of glucose transporters (GLUT 1 and GLUT 4) from the Golgi complex to the cell membrane. Likewise, ALA stimulates the activity of the insulin receptor and its substrates (IR and IRS1) as well as phosphoinositol-3-kinase (PI3K), promoting tyrosine phosphorylation in the IR and improving the glucose uptake that is dependent on PI3K. Using the above-mentioned mechanisms, the ALA is capable of attenuating the formation of advanced glycation end products by reducing the concentration of circulating glucose and preventing it from reacting with proteins with a prolonged half-life, which subsequently decreases the expression of the AGE receptor (receptor for advanced glycation end products (RAGE)) in the cell membrane [9].

In the present study, no statistically significant differences were found in HbA1c (%) after treatment in the group that consumed ALA compared to the PG and CG (*p* > 0.05), with these results being consistent with what was observed in other studies [49, 50]. However, a hypoglycemic effect has also been reported with higher doses (1200–1800 mg/day) of ALA so it has been noted that the effect on HbA1c (%) depends on the dose [37, 38]. This may explain the negative results of our study.

With respect to the antioxidant capacity of ALA, it has been pointed out that it is a powerful redox pair that is capable of neutralizing different ROS. In addition, it has the ability to restore the reduced/oxidized glutathione ratio (GSH/GSSG) by either transferring electrons directly to the GSSG for reduction or increasing the synthesis of glutathione through improving the plasma uptake of cystine to subsequently reduce it to cysteine, which is the precursor of glutathione. ALA is also able to regenerate the reduced forms of other antioxidants, such as vitamins C and E. Furthermore, ALA has the ability to chelate ionic metals and counteract their oxidizing effects, which gives it enormous antioxidant capacity [9].

Regarding the effect of ALA on the OxS markers, it has been reported that the administration of ALA at doses of 300–1200 mg/day for three to six months has a positive effect on different oxidative stress markers, such as MDA, SOD, GPx, PGF2*α*-isoprostane, and 8-hydroxy-2′-deoxyguanosine [37, 51]. Negative results have also been observed, as shown in the following studies: Sola et al. did not find statistically significant differences in the concentration of 8-isoprostane in adult patients with metabolic syndrome after treatment with 300 mg of ALA for 4 weeks compared with the placebo group [32]. Likewise, Khabbazi et al., in a study conducted in patients with renal failure, who were given 600 mg/day of ALA for eight weeks, did not observe statistically significant changes in MDA and total antioxidant status levels [47]; Sharman et al., in a trial conducted in healthy adults, who were given 600 mg/day of ALA for seven days, also found no changes in the levels of MDA, SOD, GPx, and catalase [52]; and Ahmadi et al. also found no changes in MDA with the administration of 600 mg/day of ALA for two months in hemodialysis patients [53]. In our study, no statistically significant differences were observed in the blood concentration of 8-isoprostane between the EG and the PG (*p* > 0.05). On the other hand, GPx, SOD, and SOD/GPx also showed no statistically significant changes in EG compared to PG after treatment. This may be due to the dose of ALA and/or the length of the follow-up of administration of the treatment, considering that ours was a population of older adults, so the degree of oxidative stress is greater due to the aging process associated to diabetes mellitus.

Regarding the anti-inflammatory properties of ALA, it has been indicated that it has the capacity to decrease the production of TNF-*α*, IL-1*β*, and IL-6 both in animal models and in humans. ALA acts at the level of phosphorylation of the factor inhibitor protein *κ*B (IKK) and prevents the activation and release of NF-*κ*B, which also prevents its translocation to the nucleus and the subsequent transcription of genes that direct the synthesis of proinflammatory proteins (TNF-*α*, IL-1*β*, and IL-6) [46, 47]. In this regard, a decrease in the markers of chronic inflammation has been reported in diabetic patients treated with ALA at doses of 300–600 mg/day for 3–6 months [32, 46, 47]. In contrast, we observed a significant increase (*p* < 0.05) in all proinflammatory parameters evaluated (CRP, TNF-*α*, IL-1*β*, IL-6, IL-8, and IL-10) in the GC after six months compared to the GE and GP in our study. Consistent with our findings regarding the effect of ALA on the OxS markers, our results suggest that the administration of 600 mg/day of ALA has an anti-inflammatory effect that is similar to placebo.

The multiple regression analysis allows us to demonstrate the relationship of the concentration of ALA with the markers of OxS and chronic inflammation. In this regard, although the correlation of ALA with TNF-*α*, IL-6, IL-1*β*, SOD, GPx, and 8-isoprostane was statistically significant, the effect of ALA was not sufficient to show statistically significant differences between the EG and PG in the blood concentration of these markers; therefore, our findings do not support the anti-inflammatory and antioxidant effect of ALA in older adults with type 2 diabetes mellitus with a dose of 600 mg/day of ALA for six months.

## 5. Limitations

It was not possible to randomize the three groups and initiate the investigation simultaneously. We did not accurately measure some noncontrollable variables, such as the amount and type of food consumed, because although they were provided with a guide on the type and quality of food they could consume, we are not sure of the degree of compliance. In this sense, the 24-hour dietary recalls does not guarantee the reliability of the data [19]. On the other hand, although all the participants were classified as sedentary, because they did not perform periodic physical exercise, the expenditure of kilocalories for the daily activities of each participant was different, and this was not measured in the study. Another important limitation of the study was not being able to try different doses of ALA at different lengths of the follow-up of administration of the treatment.

## 6. Conclusions

Our findings do not support the anti-inflammatory and antioxidant effect of ALA at a dose of 600 mg/day for six months in older adults with type 2 diabetes mellitus. In this regard, the exacerbating effect of diabetes mellitus and aging on oxidative stress and inflammation should be considered [6]. For this reason, the administration of doses of 1200–1800 mg/day of ALA could be useful to mitigate the oxidative stress and inflammation as well as to avoid the production of AGEs that occurs in T2DM in older adults, although the effectiveness of such higher doses must be verified through controlled clinical trials.

## Figures and Tables

**Table 1:** Body mass index and blood pressure by the study group.

Variable	Experimental (*n* = 42)		Placebo (*n* = 38)		Control (*n* = 28)	
	Baseline	Posttreatment	Baseline	Posttreatment	Baseline	Six months
Age (years)	63 ± 1		64 ± 1		66 ± 1	
BMI (kg/m^2^)	28.69 ± 0.64	28.32 ± 0.63	28.96 ± 1.03	28.83 ± 0.70	29.70 ± 0.98	29.44 ± 1.43
SBP (mmHg)	124 ± 3	126 ± 3	126 ± 3	127 ± 2	136 ± 3	136 ± 3
DBP (mmHg)	78 ± 2	77 ± 2	77 ± 2	77 ± 2	79 ± 2	81 ± 2

BMI: body mass index; SBP: systolic blood pressure; DBP: diastolic blood pressure. Repeated measure analysis of variance. *p* > 0.05.

**Table 2:** Biochemical parameters at baseline and posttreatment by the study group.

Variable	Experimental (*n* = 42)		Placebo (*n* = 38)		Control (*n* = 28)	
	Baseline	Posttreatment	Baseline	Posttreatment	Baseline	Six months
Hemoglobin (g/dL)	14.1 ± 0.3	13.8 ± 0.3	13.8 ± 0.3	13.6 ± 0.3	14.4 ± 0.3	14.7 ± 0.3
Hematocrit (%)	42 ± 1	41 ± 1	41 ± 1	40 ± 1	42 ± 1	42 ± 1
Glucose (mg/dL)	156 ± 9	147 ± 8	153 ± 7	145 ± 8	149 ± 12	159 ± 13
Urea (mg/dL)	35 ± 2	35 ± 2	34 ± 2	35 ± 2	35 ± 2	35 ± 2
Creatinine (mg/dL)	0.65 ± 0.02	0.78 ± 0.03	0.64 ± 0.02	0.82 ± 0.03	0.70 ± 0.06	0.59 ± 0.07
Uric acid (mg/dL)	4.4 ± 0.2	4.0 ± 0.2	4.8 ± 0.2	4.2 ± 0.2	4.1 ± 0.4	4.0 ± 0.2
Cholesterol (mg/dL)	180 ± 8	180 ± 9	176 ± 7	158 ± 8	198 ± 10	215 ± 8^∗^
Triglycerides (mg/dL)	152 ± 12	145 ± 12	142 ± 11	155 ± 13	150 ± 15	161 ± 14
HDL-C (mg/dL)	48 ± 1	54 ± 2	46 ± 2	51 ± 2	64 ± 2	58 ± 3^∗^
Albumin (g/dL)	4.6 ± 0.1	4.6 ± 0.1	4.5 ± 0.1	4.6 ± 0.1	4.5 ± 0.1	4.5 ± 0.1
HbA1c (%)	8.9 ± 0.2	8.6 ± 0.3	8.8 ± 0.2	8.4 ± 0.3	8.8 ± 0.3	9.1 ± 0.3

HDL-C: high-density lipoprotein cholesterol; HbA1c: glycosylated hemoglobin. The values represent mean + standard error. Repeated measure analysis of variance. Dunnett's post hoc test. ^∗^*p* < 0.05.

**Table 3:** Oxidative stress markers at baseline and posttreatment by the study group.

Markers	Experimental (*n* = 42)		Placebo (*n* = 38)		Control (*n* = 28)	
	Baseline	Posttreatment	Baseline	Posttreatment	Baseline	Six months
8-Isoprostane (pg/mL)	100 ± 3	57 ± 3	106 ± 7	77 ± 5	94 ± 10	107 ± 11^∗^
SOD (UI/L)	178 ± 1	177 ± 1	176 ± 1	172 ± 1	182 ± 2	172 ± 1^∗^
GPx (UI/L)	8409 ± 507	9694 ± 458	8273 ± 575	8691 ± 355	9531 ± 815	6223 ± 613^∗^
TAS (*μ*mol/L)	1045 ± 27	986 ± 24	1144 ± 34	951 ± 34	1107 ± 51	1060 ± 52
AOGAP (*μ*mol/L)	796 ± 20	729 ± 21	843 ± 29	702 ± 27	978 ± 49	883 ± 58
SOD/GPx	0.024 ± 0.001	0.020 ± 0.001	0.026 ± 0.002	0.021 ± 0.001	0.026 ± 0.004	0.031 ± 0.002^∗^
RAGE	1636 ± 88	1144 ± 68	1506 ± 97	1016 ± 82	1407 ± 112	1506 ± 128^∗^

SOD: superoxide dismutase; GPx: glutathione peroxidase; TAS: total antioxidant status; AOGAP: antioxidant gap; SOD/GPx: SOD/GPx ratio; RAGE: receptor for advanced glycation end products; SE: standard error. Values are the means ± SE. Repeated measure analysis of variance. Dunnett's post hoc test. ^∗^*p* < 0.05.

**Table 4:** Inflammatory markers at baseline and posttreatment by the study group.

Markers	Experimental (*n* = 42)		Placebo (*n* = 38)		Control (*n* = 28)	
	Baseline	Posttreatment	Baseline	Posttreatment	Baseline	Six months
CRP (mg/dL)	0.26 ± 0.04	0.26 ± 0.04	0.25 ± 0.04	0.29 ± 0.05	0.25 ± 0.05	0.37 ± 0.06^∗^
TNF-*α* (pg/mL)	6.56 ± 0.20	6.48 ± 0.20	6.62 ± 0.17	6.66 ± 0.16	7.99 ± 0.24	8.87 ± 0.25^∗^
IL-10 (pg/mL)	3.31 ± 0.17	3.41 ± 0.19	3.07 ± 0.09	3.16 ± 0.05	2.96 ± 0.11	3.58 ± 0.10^∗^
IL-6 (pg/mL)	5.07 ± 0.23	5.06 ± 0.21	5.27 ± 0.19	5.24 ± 0.31	5.17 ± 0.19	6.13 ± 0.17^∗^
IL-1*β* (pg/mL)	9.46 ± 0.27	9.30 ± 0.18	9.51 ± 0.20	9.45 ± 0.12	9.78 ± 0.23	10.96 ± 0.20^∗^
IL-8 (pg/mL)	16.20 ± 0.9	15.61 ± 1.0	17.20 ± 1.4	16.74 ± 1.2	15.5 ± 0.75	18.10 ± 1.57^∗^

CRP: C-reactive protein; TNF-*α*: tumor necrosis factor-alpha; IL-10: interleukin 10; IL-6: interleukin 6; IL-1*β*: interleukin 1*β*; IL-8: interleukin 8; SE: standard error. Values are the means ± SE. Repeated measure analysis of variance. Dunnett's post hoc test. ^∗^*p* < 0.05.

**Table 5:** Correlation between ALA, HbA1c, RAGE, and oxidative stress markers after six months.

	ALA	HbA1c	RAGE	SOD	GPx	TAS	AOGAP	SOD/GPx	8-ISOP
*r*									
ALA	1	0.084	-0.100	0.279	0.249	0.038	0.006	-0.180	-0.247
HbA1c		1.000	-0.020	-0.131	-0.041	0.017	0.061	-0.004	0.157
RAGE			1.000	-0.084	-0.302	-0.245	-0.069	0.290	-0.026
SOD				1.000	0.134	0.119	0.165	-0.076	-0.109
GPx					1.000	-0.060	-0.020	-0.828	-0.145
TAS						1.000	0.649	0.137	0.187
AOGAP							1.000	0.047	0.079
SOD/GPx								1.000	0.139
8-ISOP									1.000
*p*									
ALA		0.402	0.329	0.005	0.012	0.702	0.952	0.077	0.017
HbA1c			0.845	0.194	0.690	0.862	0.546	0.969	0.134
RAGE				0.415	0.003	0.014	0.504	0.005	0.810
SOD					0.186	0.233	0.103	0.457	0.303
GPx						0.546	0.841	0.000	0.170
TAS							0.000	0.177	0.068
AOGAP								0.653	0.453
SOD/GPx									0.200
8-ISOP									

ALA: alpha-lipoic acid; HbA1c: glycosylated hemoglobin; RAGE: receptor for advanced glycation end products; SOD: superoxide dismutase; GPx: glutathione peroxidase; TAS: total antioxidant status; AOGAP: antioxidant gap; SOD/GPx: SOD/GPx ratio; 8-ISOP: 8-isoprostane.

**Table 6:** Correlation between ALA and inflammatory markers after six months.

	ALA	TNF-*α*	IL-10	IL-6	IL-1*β*	IL-8	CRP
*r*							
ALA	1	-0.250	0.041	-0.249	-0.329	0.067	-0.100
TNF-*α*		1.000	0.289	0.188	0.574	0.126	0.030
IL-10			1.000	0.370	0.361	0.222	-0.010
IL-6				1.000	0.230	0.154	0.290
IL-1*β*					1.000	0.136	0.003
IL-8						1.000	0.085
CRP							1.000
*p*							
ALA		0.014	0.694	0.019	0.001	0.545	-0.100
TNF-*α*			0.005	0.076	0.000	0.249	0.775
IL-10				0.000	0.000	0.041	0.922
IL-6					0.028	0.169	0.006
IL-1*β*						0.213	0.977
IL-8							0.440
CRP							

ALA: alpha-lipoic acid; TNF-*α*: tumor necrosis factor-alpha; IL-10: interleukin 10; IL-6: interleukin 6; IL-1*β*: interleukin 1*β*; IL-8: interleukin 8; CRP: C-reactive protein.

## Data Availability

Data on biochemical, clinical, and anthropometric values (database in Excel) used to support the findings of this study are available from the corresponding author upon request.
